# The calmodulin intergenic spacer as molecular target for characterization of *Leishmania* species

**DOI:** 10.1186/1756-3305-7-35

**Published:** 2014-01-19

**Authors:** Aracelis Miranda, Franklyn Samudio, Azael Saldaña, Juan Castillo, Adeilton Brandão, Jose E Calzada

**Affiliations:** 1 Instituto Conmemorativo Gorgas de Estudios de Salud, Ciudad de Panamá, Panamá; 2 Laboratorio Interdisciplinar de Pesquisas Médicas, Instituto Oswaldo Cruz, Fiocruz, Rio de Janeiro, Brasil

**Keywords:** Leishmania, *C*almodulin, Intergenic spacer, 3′UTR, Phylogenetic analysis, Panama

## Abstract

**Background:**

Human leishmaniasis is a neglected disease caused by parasites of the genus *Leishmania*. Clinical aspects of this disease can vary significantly, reflecting the wide range of parasites in the genus *Leishmania*. Knowing accurately the *Leishmania* species infecting humans is important for clinical case management and evaluation of epidemiological risk. Calmodulin is an essential gene in trypanosomatids that modulates the calcium metabolism in various cellular activities. Despite its strong conservation in trypanosomatids, it has been recently observed that its untranslated regions (UTR) diverge among species.

**Methods:**

In this study we analyzed the sequences and the absolute dinucleotide frequency of the intergenic spacer of the calmodulin gene (containing both, 3′ and 5′UTR) in nine reference *Leishmania* species and ten clinical isolates obtained from patients with cutaneous leishmaniasis.

**Results:**

We show that the short calmodulin intergenic spacers exhibit features that make them interesting for applications in molecular characterization and phylogenetic studies of *Leishmania*. Dendrograms based on sequence alignments and on the dinucleotide frequency indicate that this particular region of calmodulin gene might be useful for species typing between the *Leishmania* and *Viannia* subgenera.

**Conclusions:**

Mutations and composition of the calmodulin intergenic spacer from *Leishmania* species might have taxonomic value as parameters to define if an isolate is identical to a certain species or belongs to one of the two current subgenera.

## Background

Human leishmaniasis is a neglected disease caused by parasites of the genus *Leishmania*. More than 350 million people are considered at risk of contracting leishmaniasis, and 2 million cases occur yearly in tropical and subtropical countries [1]. Clinical features of this disease can vary significantly, reflecting the wide range of parasites in the genus *Leishmania*. The sub-genus *Viannia* is responsible for cutaneous leishmaniasis (CL) in South and Central America, and currently five species are epidemiologically relevant in this region: *L. V. braziliensis*, *L. V. panamensis*, *L. V. peruviana*, *L. V. guyanensis, L. V. lainsoni*, *L. V. naiffi*[1,2]. These species share a number of insect vector and vertebrate hosts, and all of them can cause infections in humans that eventually may lead to disease [3]. Knowing accurately the *Leishmania* species infecting humans is important for clinical case management and evaluation of epidemiological risk. In this sense, several molecular markers have been developed for *Leishmania* species identification, strain typing, and consequent phylogenetic studies. However, no single genetic marker has been shown to have the sufficient discriminatory power when dealing with closely related *Leishmania* species. The evaluation of other genetic markers and better analytical tools are needed to completely understand the genetic complexity of *Leishmania* parasites.

The calmodulin gene is an interesting molecular marker to evaluate. This gene encodes a protein that modulates the interaction of calcium ion with several other proteins in various cellular activities and is a key gene in the metabolism of the cell [4]. In trypanosomes it was first described in *T. brucei gambiense*[5] and later in *T. cruzi*[6]*.* Interestingly*,* mutations of calmodulin intergenic spacer have been demonstrated to be specific for *T. cruzi* major groups [7]*.* In *Leishmania* species, after genome sequencing of some species available at TriTrypDB [8], calmodulin appears in the genome at 1–3 copies depending on the species. This tandem arrangement of calmodulin gene has also been observed in *T. cruzi,* and has been deployed in a PCR amplification procedure that yields fragments containing the 3′ UTR, intergenic spacer and the 5′ UTR [7].

Since the length of the intergenic spacer between calmodulin coding sequences (CDS) varies both intra and inter species, we analyzed only the shortest segment from each *Leishmania* species.

Additionally, as a tool to supplement information about the molecular taxonomy of *Leishmania*, we analyzed the composition of dinucleotide frequency from the short calmodulin intergenic spacer to characterize reference strains and clinical isolates of *L. panamensis.* We show that the overall nucleotide composition of this segment presents features that make them interesting for applications in molecular characterization of *Leishmania*.

## Methods

*Leishmania* reference strains used in this study are listed in Table 1. Promastigotes were cultured in Schneider ’s insect medium (Sigma Aldrich, Inc., St. Louis, USA) supplemented with 20% heat-inactivated fetal bovine serum (Gibco, Grand Island, USA) at 26°C. Total genomic DNA was extracted from promastigotes using a commercial kit (Wizard Genomic DNA Purification Kit, Promega, Madison, WI) according to the manufacturer’s instructions. For amplification, oligonucleotides 5utrcal (5′ GGAGATCTGCTCGTTGGACA 3′) and 3utrcal (5′ GGTCAAATCAACTACGAGGA 3′) were designed based on the open reading frame of the calmodulin gene in order to amplify the 3′ UTR, the intergenic region and the 5′ UTR [7]. PCR was carried out in a 25 μL volume containing 12.5 μL Master Mix (Promega), 0.4 μmol/L of each oligonucleotide, 1 μL MgCl_2_ 25 mM, 1 ng DNA (1 μL) and 9.5 μL distilled water. Thirty five amplification cycles of 96°C for 10 s, 55°C for 30 s and 72°C for 30 s, with a final extension at 72°C for 7 minutes were carried out. Ten μL of the amplified products were loaded onto 0.8% agarose gel in 1X TBE (89 mM Tris borate, 2 mM EDTA [pH 8.3]) and submitted to electrophoresis at 100 V for 1 h. The gel was stained with Ethidium Bromide and visualized under UV light.

**Table 1:** ***Leishmania *** **reference strains analyzed in this study**

**Species**	**International code**
*L (Viannia) panamensis*	MHOM/PA/98/WR 2306
*L (Viannia) peruviana*	MHOM/PE/05/WR 2771
*L (Viannia) braziliensis*	MHOM/PA/02/WR 2355
*L (Viannia) braziliensis*	MHOM/BR/1975/M2903
*L (Viannia) guyanensis*	MHOM/BR/1975/M4147
*L (Viannia) lainsoni*	MHOM/BR/1981/M6426
*L (Leishmania) chagasi*	MHOM/BR/1974/PP75
*L (Leishmania) amazonensis*	IFLA/br/1967/PH8
*L (Leishmania) mexicana*	MHOM/BZ/1982/BEL21

The amplified products were submitted to 1% low melting point agarose gel in 1X TBE (89 mM Tris borate, 2 mM EDTA [pH 8.3]) electrophoresis and the product band was excised from agarose gel and purified using the Qiaquick gel extraction Kit (Qiagen, Chatsworth, Calif) following the manufacturer’s instructions. For cloning, the purified PCR products were ligated into the pCR™4-TOPO vector using the TOPO TA cloning kit for sequencing (Invitrogen). The ligation product was used to transform JM109 *Escherichia coli* cells (Promega) following standard protocol [9]. Plasmids were purified with Wizard Plus SV Minipreps (Promega, USA). DNA sequencing of both strands was carried out using BigDye Terminator 3.1 cycle sequencing kit (Applied Biosystems). Primers and Deoxynucleoside Triphosphates were removed using Xterminator kit (Applied Biosystems). The clean sequencing reaction was run through an ABI 3130x sequencer. The chromatograms were edited and aligned with MEGA v.5.0 [10] and Clustal X [11]. Calmodulin intergenic spacer sequences from species listed in Table 2 were obtained from Tritryp database (http://www.tritrypdb.org). All sequences were retrieved in fasta format and analyzed with software from emboss package (http://emboss.bioinformatics.nl/) as follows: compseq - a) dinucleotide frequency, parameter =2.

**Table 2:** ***Leishmania*** **species with genome sequences available at TriTrypdb**

**Species**	**International code/strain**
*L. braziliensis*	MHOM/BR/75/M2904
*L. donovani*	BPK282A1
*L. infantum*	JPCM5
*L. major*	strain Friedlin
*L. mexicana*	MHOM/GT/2001/U1103
*L. panamensis*	MHOM/COL/81/L13

The output files containing dinucleotide frequency were submitted to webserver http://www.hiv.lanl.gov/content/sequence/HEATMAP/heatmap.html for heatmap cluster hierarchical analysis and to PAST [12] for a paired group euclidean distance cluster analysis.

The calmodulin intergenic sequences obtained from *Leishmania* reference strains were deposited in GeneBank-NCBI under accession numbers: JN966910 to JN966919, JQ302012 and JQ302013.

This molecular approach was then employed to analyze ten *Leishmania* clinical isolates obtained from patients with localized ulcerated lesions who attended the Tropical Medicine Reference Clinic at Gorgas Memorial Institute. Use of these clinical samples was approved by the National Review Board, (Comité Nacional de Bioética de la Investigación, Instituto Conmemorativo Gorgas de Estudios de la Salud, Panama City, Panama).

### Ethical approval and consent

This molecular approach was then employed to analyze ten *Leishmania* clinical isolates obtained from patients with localized ulcerated lesions who attended the Tropical Medicine Reference Clinic at Gorgas Memorial Institute. Use of these clinical samples was approved by the National Review Board, (Comité Nacional de Bioética de la Investigación, Instituto Conmemorativo Gorgas de Estudios de la Salud, Panama City, Panama). Written informed consent was obtained from the patient for the publication of this report.

## Results and discussion

**Figure 1:**
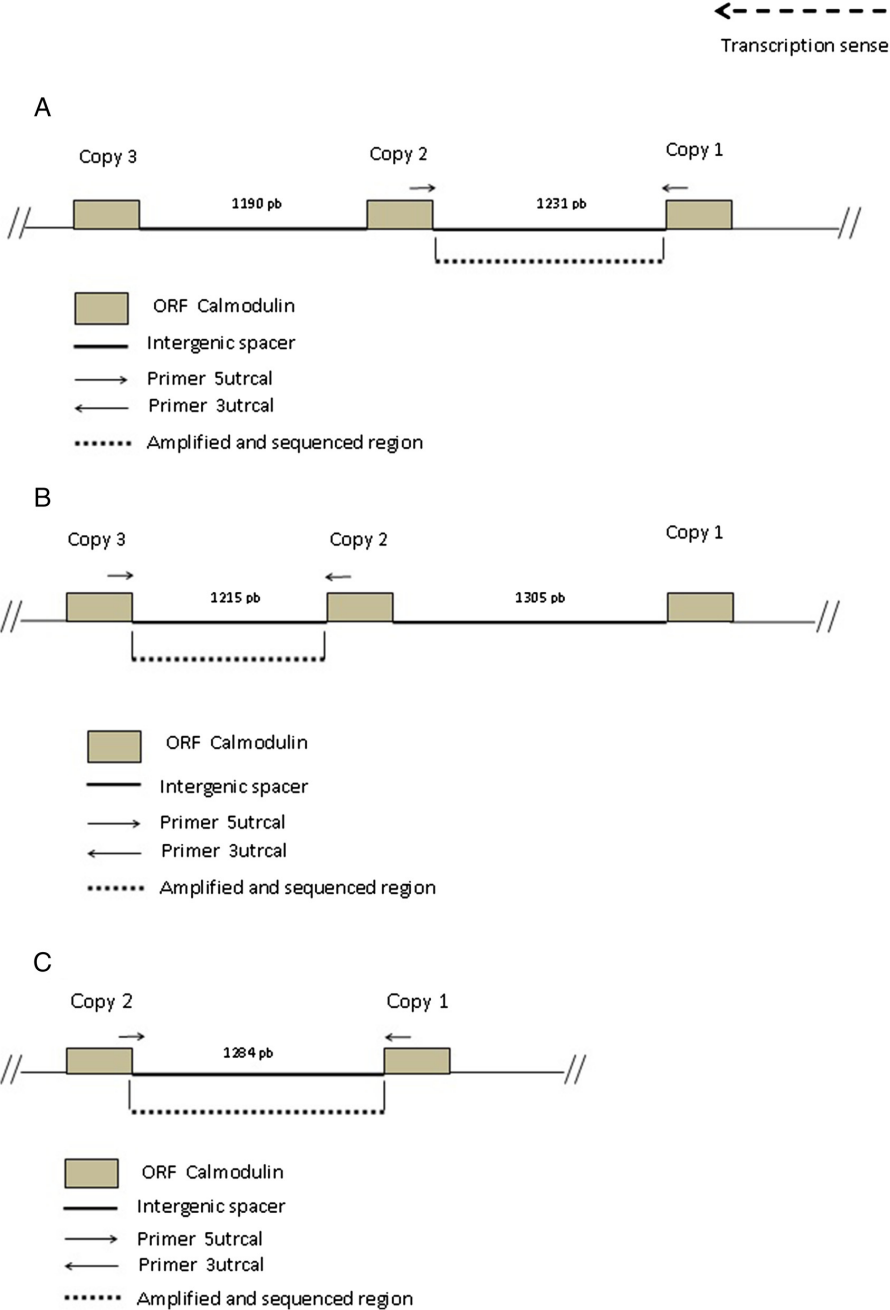
**Calmodulin gene genomic organization. (A)***Leishmania braziliensis*, **(B)***Leishmania mexicana*, **(C)***Lesihmania infantum.* Arrowheads indicate primer hybridization sites. Amplification proceeds towards intergenic spacer.

**Figure 2:**
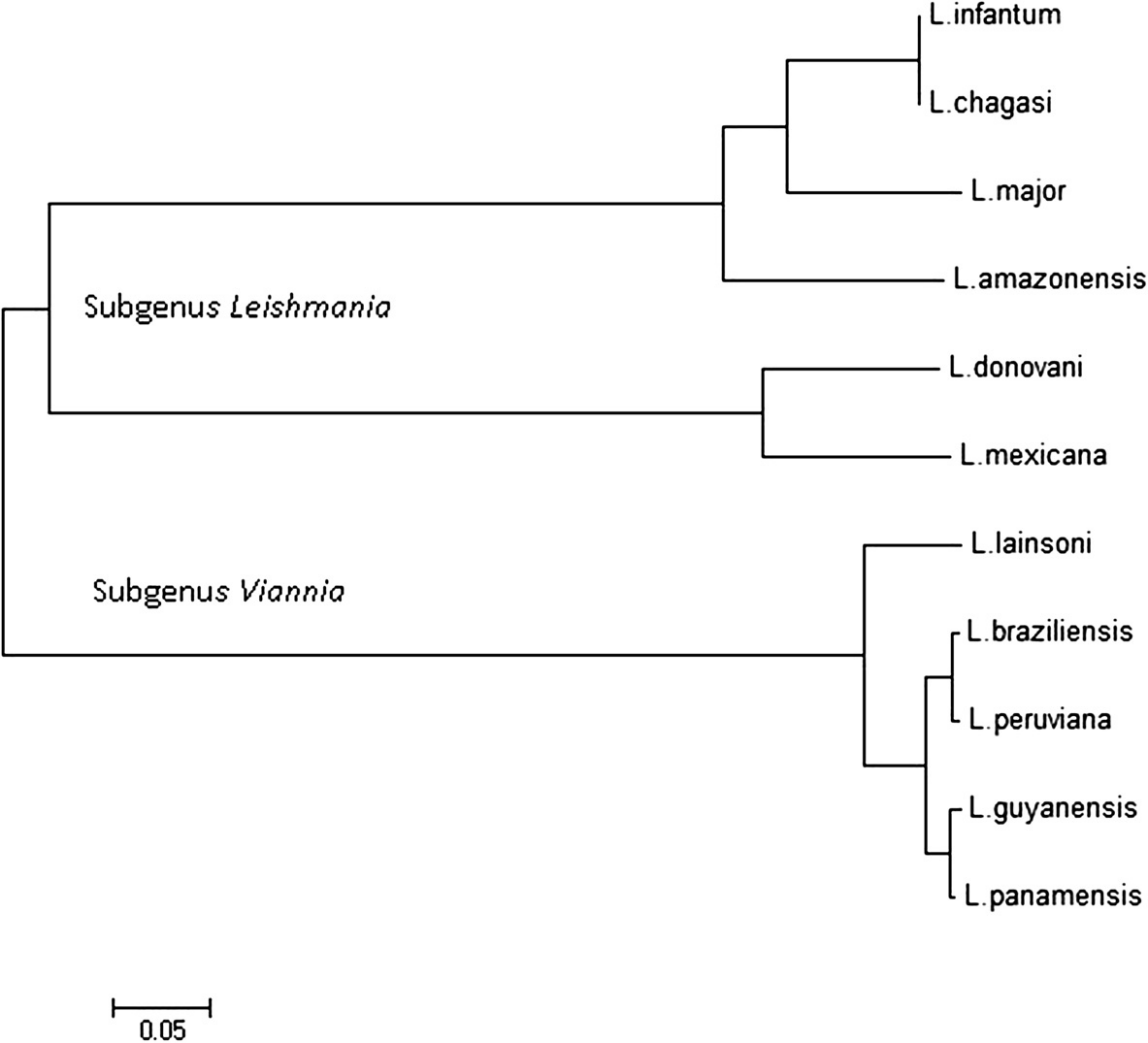
**Neighbor-joining phylogeny *Leishmania *species dendogram of alignment of calmodulin intergenic spacer sequences.** Cluster of all studied species into the two currently accepted sub-genera, *Leishmania* and *Viannia.*

A graphical summary and the PCR amplification strategy of the calmodulin locus based on genome public sequences from three *Leishmania* species (*L. braziliensis*, FR798983.1; *L. mexicana*, FR799562.1; *L. infantum*, FR796441.1) is shown in Figure 1. There is variability in both the calmodulin copy number (2–3 copies) and the length of the intergenic spacer (1,190 to 1,400 bp) among *Leishmania* species (Figure 1). We selected species for this study that have at least two calmodulin copies and analyzed the shortest segment of each species listed on Table 1. After PCR amplification and cloning, we compared their sequences with the ones available at Tritrypdb (Table 2). A simple alignment of these sequences with the ones available in GeneDB, allowed the clustering of all studied species into the two currently accepted sub-genera, *Leishmania* and *Viannia* (Figure 2). This clustering is interesting because due to low evolutionary pressures, intergenic segments may not reflect taxonomic classifications that are grounded on biological and/or clinical parameters, even for a highly conserved gene like calmodulin.

**Figure 3:**
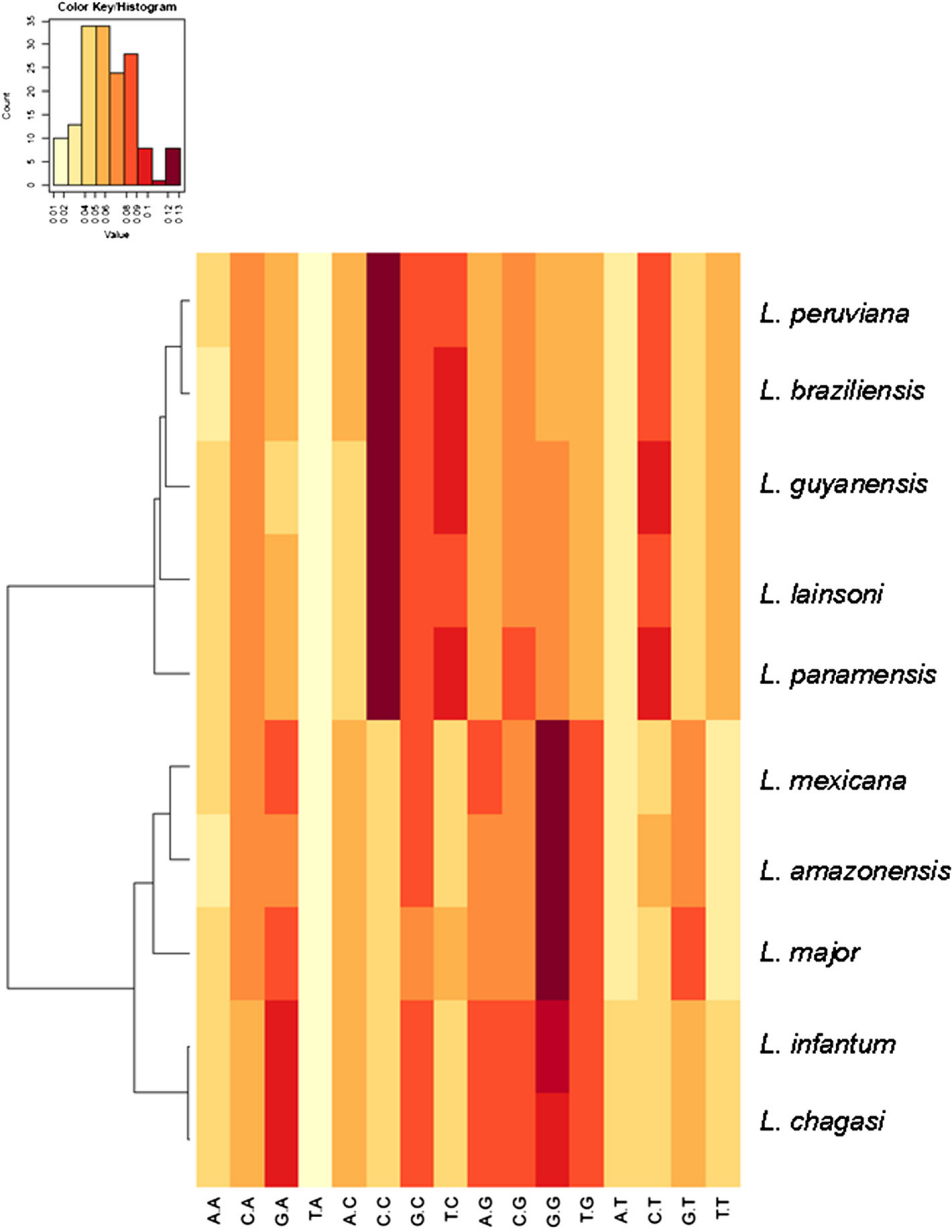
***Leishmania *species heat map diagram display of the intergenic spacer composition.** Is represented by the frequency distribution of the sixteen dinucleotides. Species are clustered according to allocation in subgenus *Leishmania* or *Viannia*.

We next used a bioinformatics tool to further evaluate the distinctiveness among these species taking into account the nucleotide composition, regardless of base order and length. We extracted from the whole intergenic segment from each species the absolute frequency of all sixteen dinucleotides. This approach based on the absolute dinucleotide frequency has recently been used to analyze the composition of trypanosomatid kDNA minicircles [13]. Figure 3 displays a graphical result of this analysis. Again, species are clustered according to allocation in subgenus *Leishmania* or *Viannia*. A large proportion of the dinucleotide composition of the calmodulin spacer is represented by CC in sub-genus *Viannia* and by GG in sub-genus *Leishmania*.

**Figure 4:**
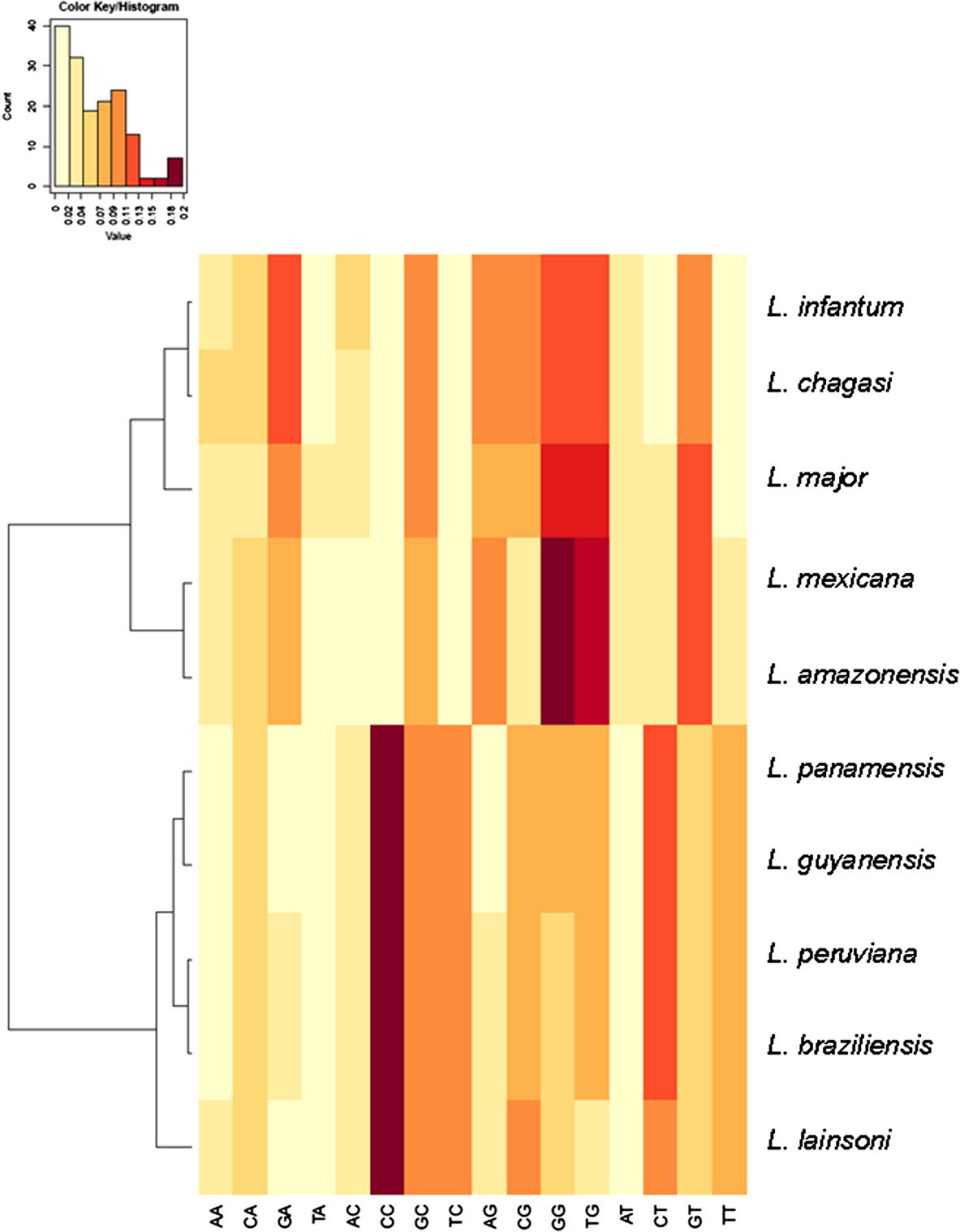
***Leishmania *species heat map of 5′ UTR calmodulin intergenic spacer composition of first 100 bp.** Species are clustered according to allocation in subgenus *Leishmania* or *Viannia*.

**Figure 5:**
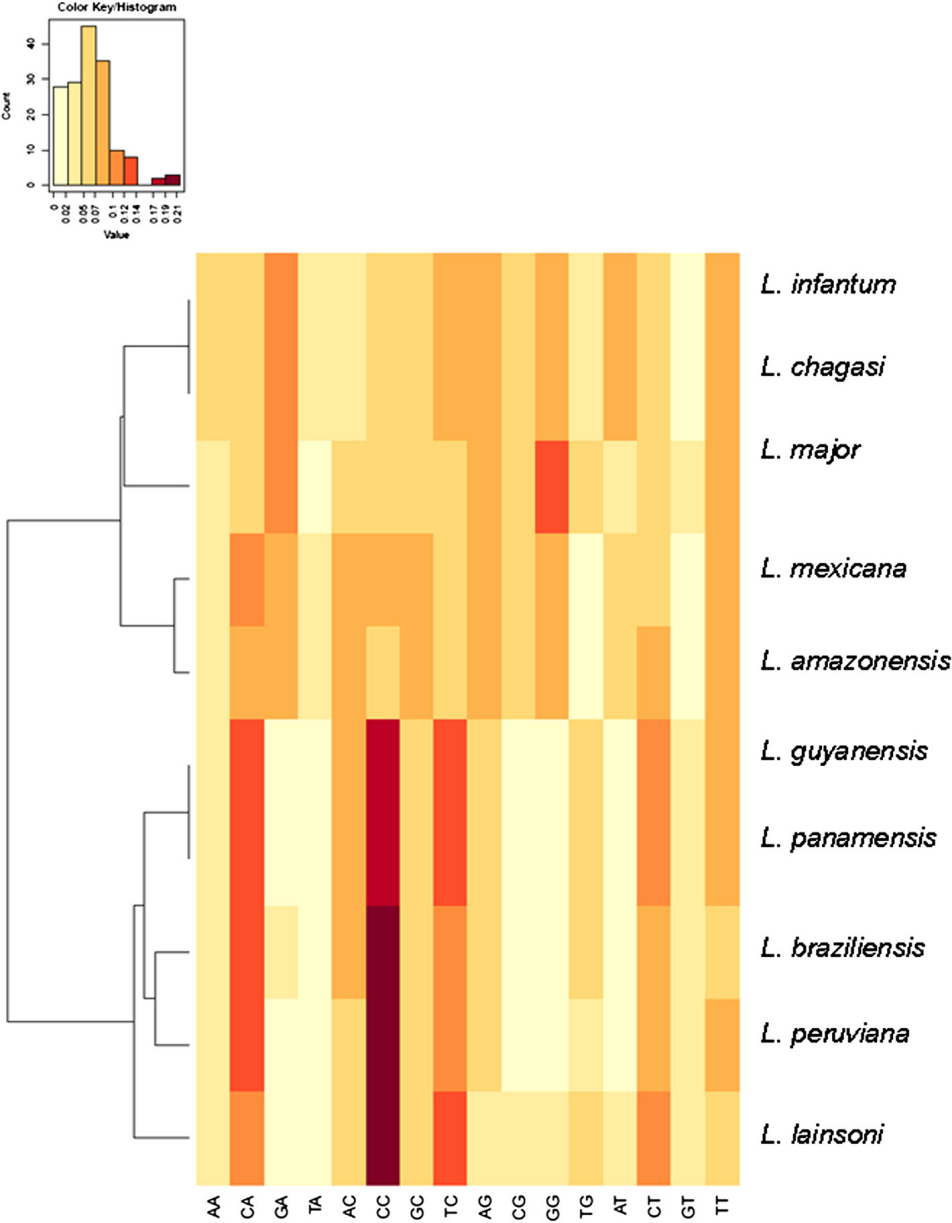
***Leishmania *species heat map of 3′ UTR calmodulin intergenic spacer composition of last 300 bp.** Species are clustered according to allocation in subgenus *Leishmania* or *Viannia*.

Since this intergenic segment includes both UTRs, (3′ and 5′UTR), we then analyzed the nucleotide composition for each UTR separately. Currently, the genome annotation does not indicate where the 5′ UTR starts or 3′ UTR ends. Thus, we assume an average of 100 and 300 bp for the 5′ and 3′ UTR, respectively. Figures 4 and 5 show this compositional analysis expressed in a heatmap. There is some bias to pyrimidine in these probable UTR containing segments, as the compositional picture does not differ from the whole segment analysis, leading to the same dichotomy into two subgenera. Besides confirming the subgenus dichotomy, this method allows a more precise characterization of species as demonstrated by the distinct branch in the distance dendrogram.

**Figure 6:**
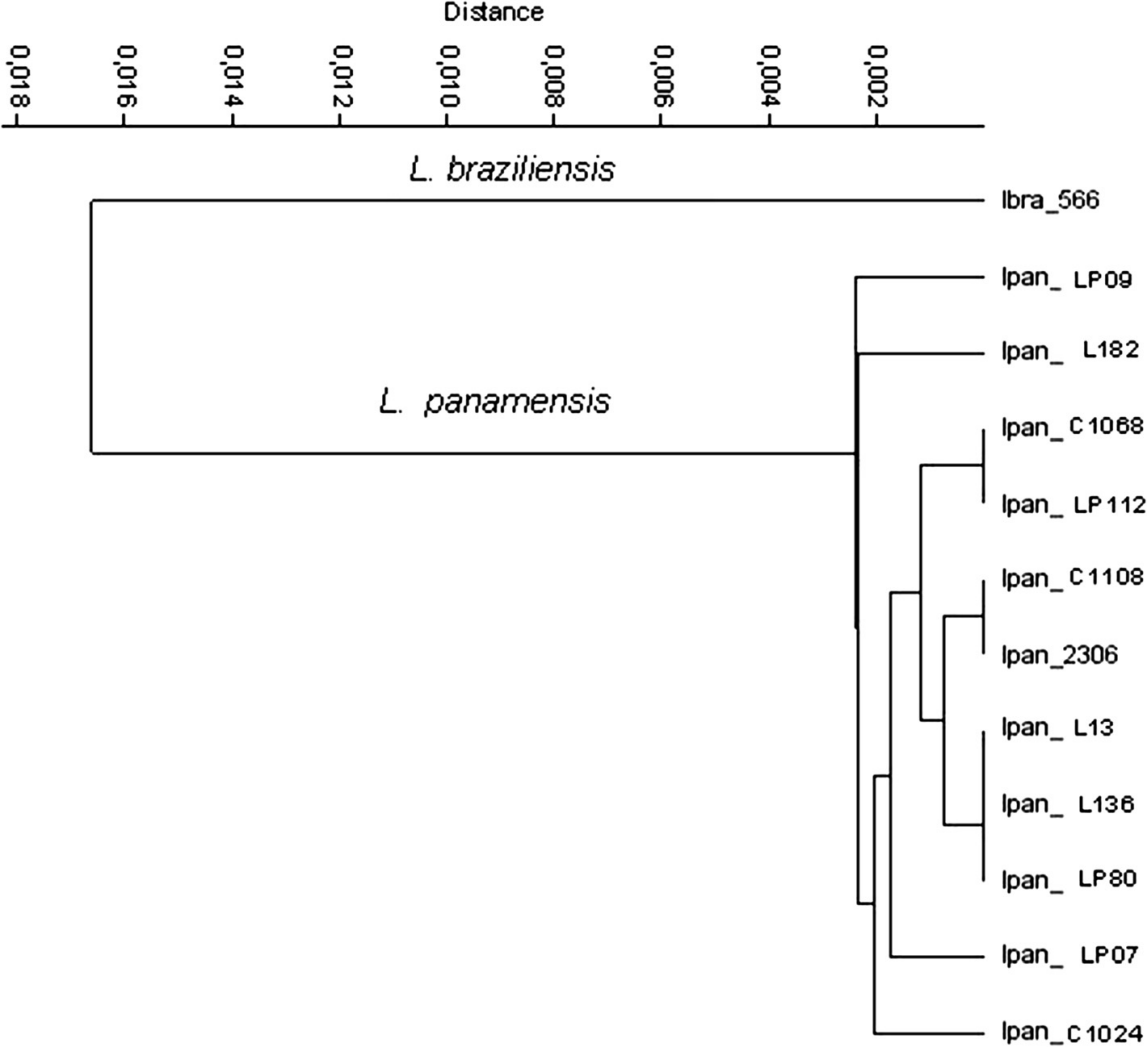
***Leishmania panamensis *isolates from Panamanian patients.** Cluster analysis confirms that all isolates belong to *L. panamensis*. Sequence accession number LP09: JN966931, L182: JN966927, C1068: JN966921, LP 112: JN966935, C1108: JN966921, L13: JN966925, L136: JN966926, LP80: JN966933, LP07: JN966929, C1024: JN966920.

In order to deploy the compositional profile of this segment for field applications, we chose the absolute dinucleotide frequency for characterization of *L. panamensis* clinical isolates. As demonstrated in Figure 6, this method worked well for the purpose of species/strain typing, as minor differences in composition allows discrimination of the isolates in comparison to reference strains (*L. panamensis* 2306 and *L. braziliensis* 566). These isolates were also characterized at Gorgas Institute as *L. panamensis* through clinical parameters and biochemical methods. The obtained compositional profiles and subsequent cluster analysis confirm that all field isolates belong to *L. panamensis*.

The *Leishmania* genus has long been considered a puzzle from the taxonomical point of view. Several clustering schemes based on clinical and biological/biochemical parameters have been proposed to better describe the species spectrum in this genus [14-16]. However, despite enormous progress on the molecular methods, including genomics and proteomics, to shed light on taxonomy and improve the *Leishmania* species characterization, doubts on the phylogenetic status of certain species still challenge the researchers on this field [17]. Despite the strong conservation of calmodulin gene in trypanosomatids, its UTRs diverge among species. For example, inspection of genome sequences containing the calmodulin 5′and 3′ UTR from *T. brucei* are different from the corresponding ones in *T. cruzi*, *L. major* and *L. braziliensis* (a blast search at http://www.tritrypdb.org shows this fact). However, this divergence is not free of evolutionary or functional pressures because the spacer and UTRs of calmodulin gene are part of a gene class that undergoes polycistronic transcription [18]. As the goal in the investigation of *Leishmania* species diversity is also to develop markers that can pick up the major transitions in the population structure, calmodulin intergenic segments are good targets for such analysis. Though intraspecies sequence conservation is observed, subgenus *Viannia* species are clearly distinct from the subgenus *Leishmania* ones in both length and nucleotide composition. However, taking into account that trypanosomatid gene expression is dependent on post transcriptional processes that are certainly controlled by elements in the 5′ and 3′ UTR and spacer (polypyrimidines region, for instance) [19-21] we decided to inspect these segments with tools based on dinucleotide composition. Mutations and composition of the intergenic segments for some of the *Leishmania* species analyzed here might have taxonomic value as parameters to define if an isolate is identical to a certain species or belongs to one of the two current subgenera. However, for species causing CL, as *L. braziliensis*, *L. peruviana*, and *L. panamensis* sequences are highly similar, casting doubts on the current status of these organisms as unique species.

From an epidemiological point of view it is important to know which *Leishmania* species circulate in a geographic area. However, from a medical perspective the distinction between *Leishmania* species causing human infection might be more relevant because the choice of treatment strategy is based on geographical location and mainly in the infecting species [22]. This is the case of *L. braziliensis* and *L. peruviana.* Both species co-circulate in some regions of Peru, but only *L. braziliensis* can potentially cause severe mucocutaneous complications [23]. We observed several sequence variations in the calmodulin intergenic spacer that allow a distinction between these two closely related species belonging to the *Viannia* sub-genus (Additional file 1).

Recent pioneering studies have used different regions of the heat-shock protein 70 gene (hsp70) with different molecular approaches (sequencing, RFLP, AFLP) for *Leishmania* species identification as well as for taxonomic and phylogenetic analysis [24-30]. Indeed, the hsp70 gene is probably the single-locus molecular marker more widely used for identifying/typing *Leishmania*. In particular, the hsp70/RFLP approach presents several advantages over other molecular methodologies and is now routinely used in many laboratories for these purposes.

In general, the dendrograms we obtained with calmodulin gene agrees with the ones produced using hsp70 as marker. A clear separation between the sub-genus *Leishmania* and *Viannia* was observed and nucleotide sequence variations in this segment of the gene made possible to discriminate between *Leishmania* species/complex (Figures 2, 3, 4 and 5 and Additional file 1). However, we recognize that so far we have only evaluated a small number of reference strains and field isolates. In this regard, the real discriminatory power of calmodulin as a marker and its potential phylogenetic inferences still need to be evaluated using a larger number of reference strains and validated with a more field isolates from different geographical areas.

## Conclusions

In conclusion, for the first time we have employed the calmodulin intergenic spacer sequences in a phylogenetic study of *Leishmania* species. We demonstrated that despite the evolutionary conservation of the calmodulin gene, its intergenic spacer region may be useful to help solve taxonomical questions in the *Leishmania* genus and also to characterize clinical/field isolates of a particular species.

## Abbreviations

CL: Cutaneous leishmaniasis; CDS: Coding DNA sequence; UTR: Untranslated regions.

## Competing interests

All authors declare no competing interests. AS and JEC are members of the Sistema Nacional de Investigación (SNI), SENACYT, Panama.

## Authors’ contributions

AB, JEC and AS conceived the study and participated in its design and coordination. AM, FS, AB and JC carried out the molecular genetic studies and participated in the sequence alignment and interpretation. AB, AM and JEC drafted the manuscript. All authors read and approved the final manuscript.

## Supplementary Material

Additional file 1 **Comparison of mutations and their localizations in the calmodulin intergenic spacer of *Leishmania Viannia *** **reference strains.** Click here for file
