# Supplementary material for: The calmodulin intergenic spacer as molecular target for characterization of Leishmania species
Source: Parasit Vectors. 2014 Jan 19;7:35. doi: 10.1186/1756-3305-7-35 (PMC4021611; doi:10.1186/1756-3305-7-35)
Supplement: Additional file 1 — Comparison of mutations and their localizations in the calmodulin intergenic spacer of Leishmania Viannia reference strains. [file 1756-3305-7-35-S1.pdf]

pb	Mutation
20	Substitution of a G by a A in *L. guyanensis*
163-249	In 163 pb substitution of C by T in *L. braziliensis* and *L. peruviana*<br>In 165 pb substitution of C by T in *L. lainsoni*.<br>In 179 pb substitution of T by C in *L. lainsoni*<br>In 189 pb substitution of C by T in *L. lainsoni*, *L. braziliensis* and *L. peruviana*<br>In 191 pb substitution of C by A in *L. lainsoni*<br>In 192 pb substitution of C by T in *L. lainsoni*<br>In 201 pb insertion of A *in L. braziliensis* and *L. peruviana*<br>From 204 to 218 pb deletion of 15 pb in *L. lainsoni*<br>In 220,223 and 226 pb substitution of G by C in *L. lainsoni*<br>In 224,230 and 237 pb substitution of G by A in *L. braziliensis* and *L. peruviana*<br>In 229 pb substitution of G by A in *L.lainsoni*<br>In 249 pb substitution of C by T in *L.panamensis*
271-273	In 271 pb substitution of C by A in *L. lainsoni*<br>In 273 pb substitution of T by C in *L. lainsoni*, *L. braziliensis* and *L. peruviana*
373-410	Deletion of four pb from 375 to 378 pb in *L. lainsoni* and *L. guyanensis*<br>Deletion of 377 pb and 378 in *L. braziliensis*<br>Deletion of six pb from 373 pb to 378 pb in *L. panamensis*<br>In 387 pb substitution of G by A in *L. guyanensis*<br>In 408 and 410 pb substitution of G by A in *L.lainsoni*<br>In 409 pb substitution of A by G in *L.lainsoni*
434-442	Insertion of nine pb in *L.lainsoni*
567-571	In 567 pb substitution of C by T in *L.guyanensis*<br>In 569 pb substitution of C by A in *L. lainsoni, L. braziliensis* and *L. peruviana*<br>In 570 pb substitution of C by T in *L. lainsoni*<br>In 571 pb substitution of G by A in *L. lainsoni*
744	Substitution of T by C in *L.guyanensis*
970-976	In 970 pb substitution of C by T in *L. lainsoni*, *L. braziliensis* and *L. peruviana*<br>In 971 pb substitution of G by A in *L.lainsoni*<br>In 972 pb substitution of A by T in *L.lainsoni*<br>In 976 pb substitution of C by A in *L.lainsoni*
1187-1222	In 1187 pb substitution of C by T in *L. guyanensis and L. panamensis*<br>In 1198 pb substitution of C by A in L. peruviana<br>In 1199 and 1200 pb substitution of C by T in *L.peruviana*<br>In 1201 pb substitution of C by T in *L. lainsoni*, *L. braziliensis* and *L. peruviana*<br>In 1206 and 1207 pb substitution of T by C in *L. lainsoni*, *L. braziliensis* and *L. peruviana*<br>Deletion of six pb from 1211 to 1216 pb in *L. braziliensis*<br>Deletion of 1215 pb in *L. lainsoni*<br>Insertion of four pb from 1211 to 1214 pb in *L. peruviana*<br>In 1222 susbtitution of a A by a G *in L.braziliensis*
